# False Localizing Signs in High Lumbar Stenosis: L2/3 Compression Mimicking L5 Radiculopathy

**DOI:** 10.7759/cureus.103098

**Published:** 2026-02-06

**Authors:** Mikhail De Santos, Marnie Leach

**Affiliations:** 1 Neurosurgery/Spine Surgery, Leeds Teaching Hospitals NHS Trust, Leeds, GBR

**Keywords:** electromyographic (emg), emg-ncv, false localising signs, foot drop, high lumbar stenosis, lumbar spinal stenosis (lss), minimal invasive spine surgery, motor radiculopathy, nerve conduction velocity (ncv), surgery spine

## Abstract

Degenerative lumbar spinal stenosis typically manifests with symptoms that correlate precisely to the anatomical level of compression. However, ‘false localising signs’ do exist, which can lead to diagnostic conundrums. A foot drop is classically attributed to a pathology at the L4/5 level affecting the L5 nerve root; however, it may rarely result from more proximal lumbar compressive pathologies. We report a rare case of high lumbar stenosis (L2/3) manifesting with typical and atypical symptoms, highlighting the importance of sound clinical decision-making while recognising this confounding clinical entity. A 55-year-old man presented with chronic back pain, neurogenic claudication, and a right-sided foot drop (Medical Research Council grade 1/5). Magnetic resonance imaging (MRI) of the lumbar spine revealed severe stenosis at the L2/3 level but no significant compression at the L4/5 or L5/S1 levels. Interestingly, neurophysiological studies confirmed L5 radiculopathy. The patient subsequently underwent an L2/3 decompression for neurogenic claudication. Post-operatively, the patient reported improvement in claudication and complete resolution of the foot drop, which remained stable at 12 months' follow-up. This case highlights that high lumbar lesions can mimic distal lumbosacral radiculopathy. This case emphasises that the level of the clinical deficit does not always correlate with the level of compression, leading to false localising signs. It is hypothesised that the L5 nerve root is particularly vulnerable at the L2/3 level due to its peripheral position within the thecal sac as it descends the cauda equina. Clinicians should be aware of this phenomenon to avoid unnecessary or incorrect surgical targeting and to aid in counselling and expectation setting.

## Introduction

Degenerative lumbar spinal stenosis is one of the great clinical masqueraders. Typically, it manifests as a spectrum of symptoms, including neurogenic claudication, sensory dysfunction, motor deficit or cauda equina syndrome, correlating to the anatomical level of compression [1]. We present a case of a 55-year-old male patient who displayed neurogenic claudication and a right foot drop, demonstrating a notable clinical-radiological discrepancy. This case illustrates a “false localising sign”, wherein high lumbar stenosis (L2/3) resulted in an L5 deficit. While intracranial causes are well-recognised false localisers, high lumbar spinal causes are frequently overlooked in the differential diagnosis of distal deficits [2]. We further provide a review of the literature and potential pathophysiological mechanisms behind this phenomenon and its implications for surgical planning. 

## Case presentation

**Figure 1:**
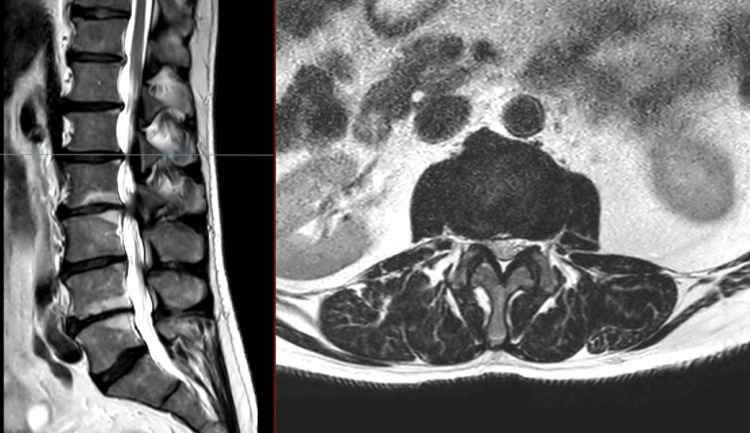
T2 MRI of the lumbar spine: sagittal (left), axial (right) at L1/2 No evidence of spinal stenosis, no compression of cauda equina were noted.

**Figure 2:**
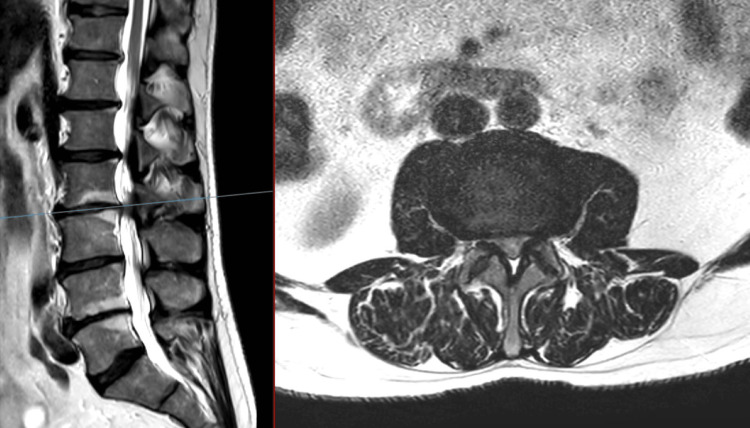
T2 MRI of the lumbar spine: sagittal (left) and axial (right) at L2/3 Moderate central spinal stenosis was noted, along with severe lateral recess stenosis. Effacement of the CSF and compression of the cauda equina were seen. Bilateral facet hypertrophy (right > left) was noted. CSF: cerebrospinal fluid

**Figure 3:**
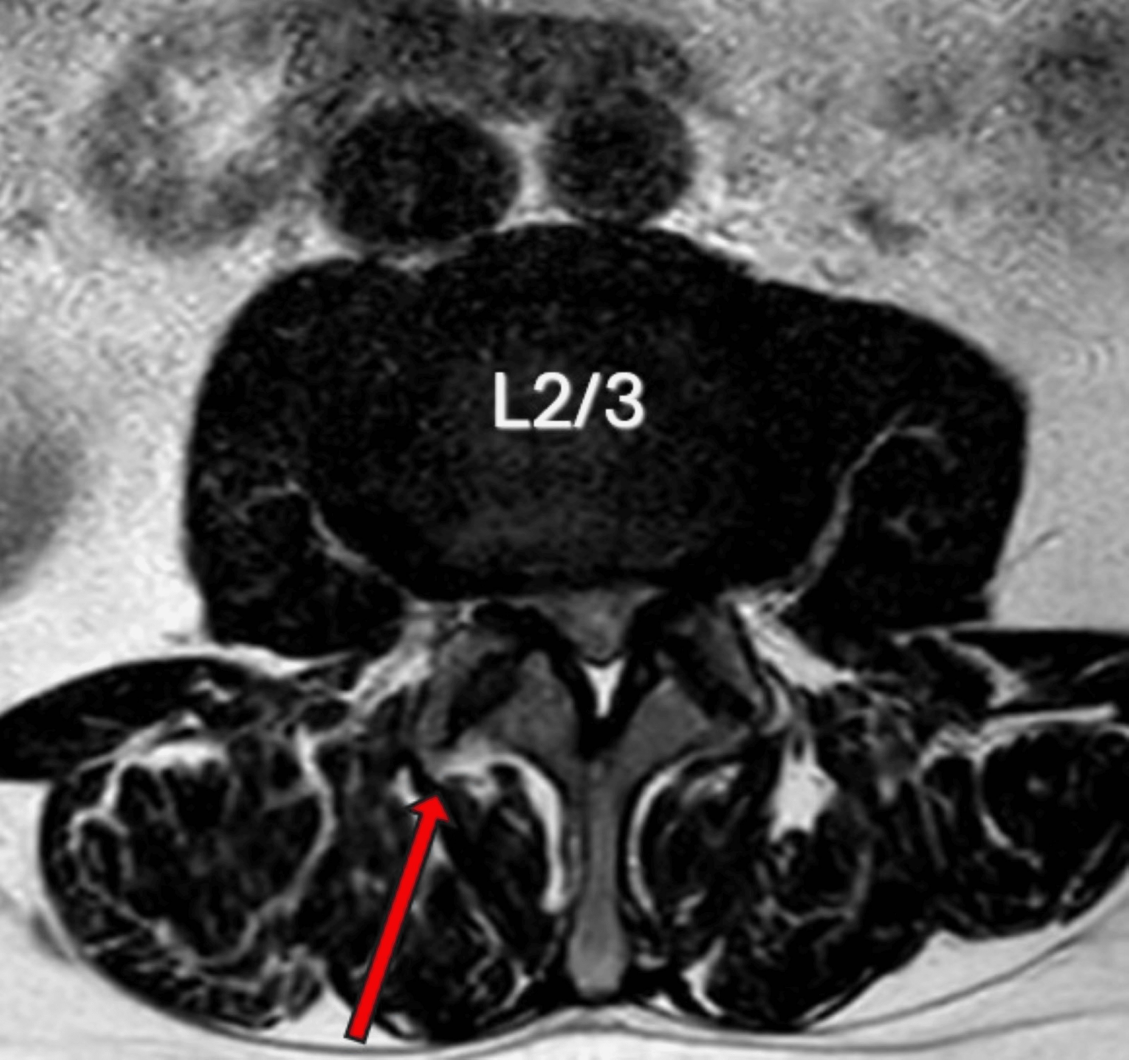
Axial T2 MRI of the lumbar spine (L2/3) demonstrating significant degenerative lumbar stenosis secondary to bilateral facet hypertrophy (R > L) with significant compression of the lateral recesses bilaterally (R > L). The red arrow demonstrates the right-sided approach used for the minimally invasive unilateral laminotomy and bilateral decompression and also highlights the side of the foot drop. R: right; L: left

**Figure 4:**
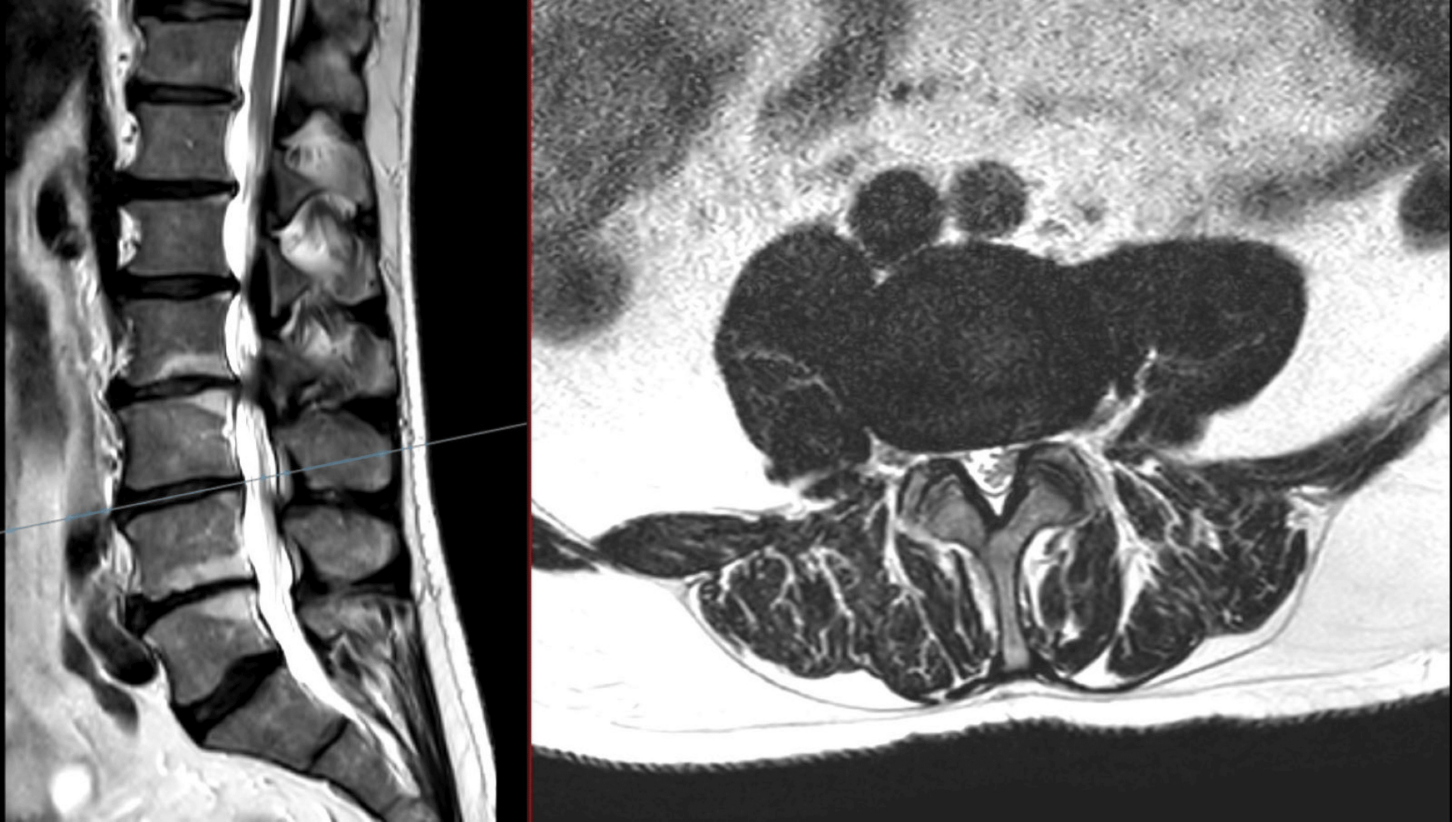
T2 MRI of the lumbar spine: sagittal (left), axial (right) at L3/4 No evidence of spinal stenosis and compression of the cauda equina was noted.

A 55-year-old man with no known co-morbidities presented to the Accident and Emergency (A&E) department with new-onset urinary dysfunction on a background of chronic lower back pain and difficulty walking. His urinary symptoms were characterised by urgency without retention or incontinence. An acute magnetic resonance imaging (MRI) scan of the lumbar spine was performed, which ruled out cauda equina syndrome (Figures 1-4). The scan demonstrated moderate stenosis at the L2/3 level but no acute compression requiring emergency intervention. Following this, the patient was referred to our spinal outpatient clinic for further management.

Upon review in the clinic, the patient reported a one-year history of lower back pain exacerbated by movement, accompanied by bilateral anterior thigh paraesthesia radiating to both shins. Significantly, he reported a six-month history of a painless right foot drop and frequent tripping, which had progressively impaired his gait. He also reported decreased sensation along the L5 dermatome. These symptoms persisted despite a trial of conservative management, including neuropathic analgesia and physiotherapy. His urinary urgency had remained stable since his initial presentation to A&E.

On physical examination, the patient exhibited a specific motor deficit with Medical Research Council (MRC) grade 1/5 power in the right extensor hallucis longus (EHL) and grade 3/5 in the tibialis anterior. Sensory examination revealed mild hypoesthesia in the right L5 dermatome. Notably, provocative testing demonstrated positive bilateral femoral stretch tests, while the bilateral Lasegue or straight leg test was negative. The remainder of the lower limb neurological examination, including tone and reflexes, was unremarkable.

**Figure 5:**
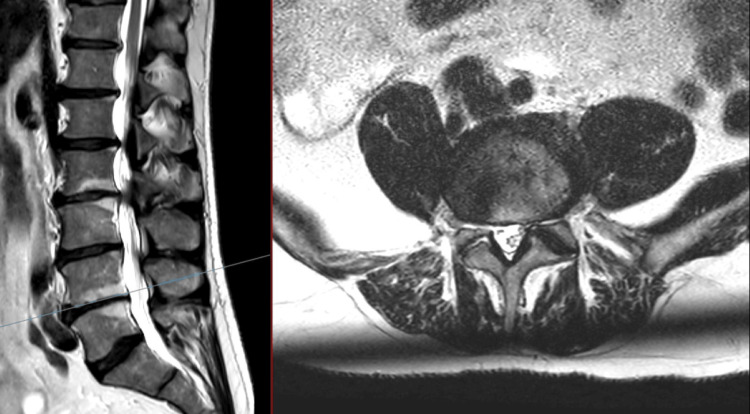
T2 MRI of the lumbar spine: sagittal (left) and axial (right) at L4/5 No compression of the cauda equina was noted. There was no evidence of compression of the right L5 nerve root.

**Figure 6:**
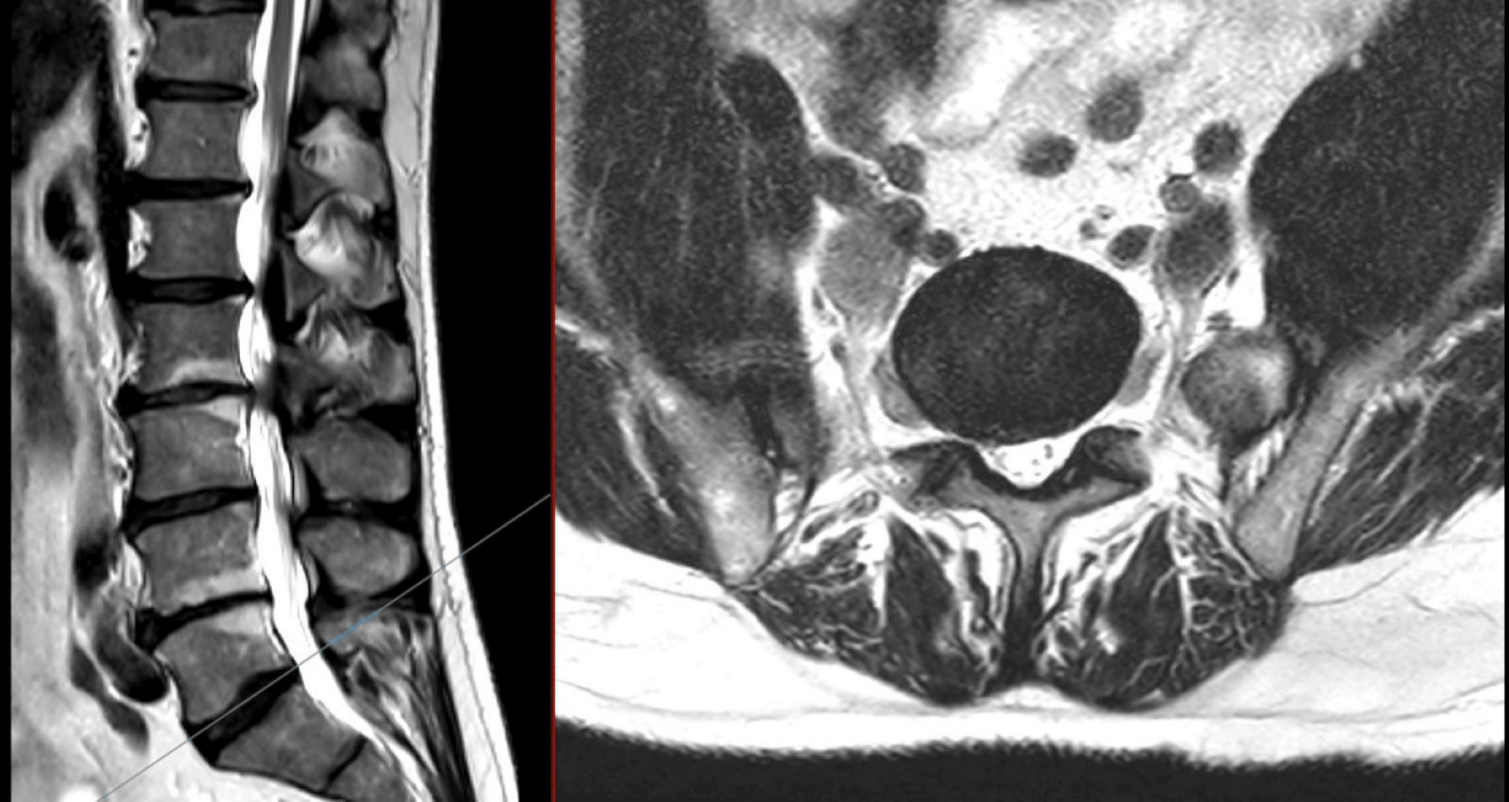
T2 MRI of the lumbar spine: sagittal (left) and axial (right) at L5/S1 Neither compression of the S1 nerve roots nor cauda equina nerve roots at this level was present.

**Figure 7:**
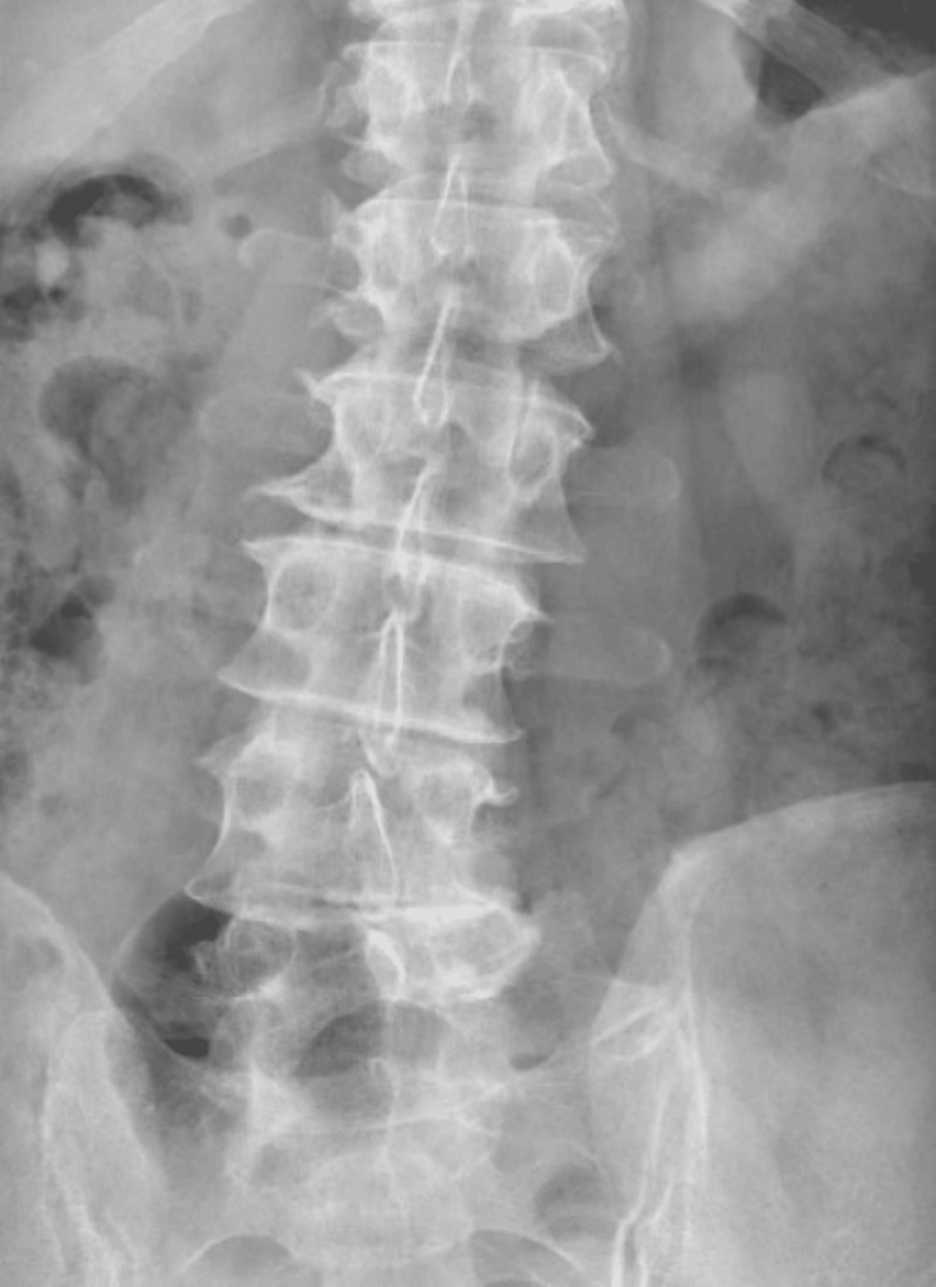
Anteroposterior lumbar X-ray demonstrating degenerative lumbar scoliosis

A detailed review of the MRI and plain radiographs of the lumbar spine revealed a mild degenerative lumbar scoliosis (apex at L4/5). Crucially, while there was central and lateral stenosis at L2/3 secondary to facet hypertrophy and a broad-based disc The caudal levels (L4/5) did not have any evidence of significant stenosis (Figures 1-7). To exclude a central cause for the foot drop, intracranial imaging was performed and found to be normal.

A clinical diagnosis of neurogenic claudication secondary to degenerative lumbar stenosis at L2/3 was made. However, a diagnostic discordance existed between the clinical findings (suggestive of L5 radiculopathy) and the radiological findings (L2/3 compression). Consequently, electromyographic (EMG) studies (Tables 1, 2) were performed to ascertain the aetiology of the right foot drop. Needle EMG examination of the L5 innervated muscles demonstrated chronic partial denervation, while the sensory nerve action potentials (SNAPs) were preserved. No active denervation was noted to suggest an acute or active pathology. Thus, these findings were suggestive of a pre-ganglionic lesion of the right L5 nerve root, supporting the diagnosis of a chronic right L5 radiculopathy.

**Table 1:** Sensory nerve conduction study of sural and peroneal nerves bilaterally within normal limits R: right; L: left; CV: conduction velocity

Sensory Nerve Conduction Study Data				
Nerve/Sites	Lat1 ms	Ampl. C μV	Dist mm	CV m/s
R Sural: antidromic				
Calf	1.9	9.6	90	48
L Sural: antidromic				
Calf	2	12.8	110	54
R Superficial peroneal				
Leg	2	7.5	110	56
L Superficial peroneal				
Leg	2.3	8.9	110	47

**Table 2:** Needle electromyographic (EMG) examination of the right limb muscles demonstrated no signs of active denervation. Chronic denervation seen in some L5 muscles. Findings suggestive of a proximal pre-ganglionic lesion of the right L5. R: right; Fib: fibrillations; PSW: positive sharp waves; MUAP: motor unit action potential; Amp: amplitude; Dur: duration ; PPP: polyphasic potentials/phases

EMG Summary Table										
Muscle	Nerve	Roots	Spontaneous: Fib	Spontaneous: PSW	Spontaneous: Other	MUAP: Amp	MUAP: Dur.	MUAP: PPP	Recruitment: Pattern	Recruitment: Firing Freq.
R. Extensor hallucis longus	Deep peroneal (fibular)	L5-S1	None	None	None	1+	1+	2+	Reduced	Normal
R. Peroneus longus	Perineal	L5-S1	None	None	None	N	N	N	N	Normal
R. Tibialis posterior	Tibial	L4-L5	None	None	None	1+	N	1+	Reduced	Normal
R. Vastus medialis	Femoral	L2-L4	None	None	None	N	N	N	N	Normal
R. Rectus femoris	Femoral	L2-L4	None	None	None	N	N	N	N	Normal
R. Gluteus medius	Superior gluteal	L4-S1	None	None	None	1+	N	1+	Reduced	Normal

Given the mismatch between the anatomical level of stenosis (L2/3) and the involved nerve root (L5), a thorough preoperative discussion was held with the patient. The possibility that the foot drop might not improve following decompression at a higher level was explicitly raised. The patient subsequently consented to and underwent an elective right-sided minimally invasive L2/3 unilateral laminotomy and bilateral decompression at L2/3 and bilateral undercutting facetectomies.

The procedure was uneventful. The patient’s immediate postoperative recovery was remarkable; neurological examination in the recovery room revealed complete resolution of the right foot drop, with power returning to MRC grade 5/5. Sensation along the L5 dermatome had also improved. He was mobilised and discharged on the same day. Subsequent outpatient follow-up at two weeks and six weeks confirmed an uncomplicated postoperative course with sustained resolution of the right ankle weakness and overall improvement in his symptoms at 12 months' follow-up.

## Discussion

The presence of false localising signs frequently challenges the clinicoanatomical paradigm, which serves as the fundamental basis for neurological assessment [2]. Classically, a foot drop, which is the hallmark of an L5 nerve root lesion, is characterised by weakness of the tibialis anterior, EHL, and extensor digitorum brevis. This clinically manifests as an impairment in ankle dorsiflexion and eversion [3]. While this typically results from a pathology at the L4/5 disc level or within the L5 neural foramen, a foot drop can rarely manifest as an upper motor neuron sign resulting from intracranial pathology, such as a stroke in the posterior limb of the internal capsule or intracranial tumours such as a parasagittal meningioma, in which case hyperreflexia is present [4]. However, as demonstrated in this case, high lumbar spine compression is a rare but critical cause of this presentation.

Several case reports (Table 3) have described high lumbar pathology causing an isolated foot drop, though these are usually the result of a herniated intervertebral disc at single or multiple levels [5,6]. Iizuka et al. analysed the possible mechanisms for the foot drop, especially as it related to lumbar spinal stenosis, but due to the multi-spinal-level involvement and, in the majority of cases, more than one level decompressed, the possible mechanism is somewhat guarded [7]. Only one other case report describes a foot drop secondary exclusively to degenerative lumbar spinal stenosis at L1/2 and L2/3 [8]. The mechanism of this false localising sign is not fully understood, but several theories have been postulated. These included Porter’s hypothesis of ‘double space compression’ resulting in venous congestion. While venous congestion could explain the pathology in Hidalgo’s report [8], our case only involved a single level of compression (L2/3).

**Table 3:** Case report (literature review) describing high degenerative lumbar spine stenosis causing foot drop. All other studies included a mixture of disc herniation or multiple-level stenosis. Neither of which was present in our case.

Author	Deficit	Levels Involved
Hidalgo-Ovejero et al. [8]	Foot drop	L1/2 and L2/3
Iizuka et al. [7]	Foot drop	L2/3, L3/4, L4/5, L5/S1

Another possible mechanism is the specific topography of the L5 nerve as it descends within the cauda equina, making it susceptible to proximal compression [6]. Cadaveric studies of the cauda equina support this theory, identifying a consistent cross-sectional pattern regarding the intrathecal nerve root paths. Lumbar and first sacral roots exhibited an oblique layered pattern as they ascended, with the smaller lower sacral and coccygeal roots located dorsally. This ventral placement of the motor bundle is held in place by invaginations of the arachnoid and is situated anteromedially to the respective sensory bundle. This is the likely explanation for the clinical presentation in this case [9].

Our case is particularly noteworthy because the patient presented with classic neurogenic claudication and a positive femoral stretch test, which pointed towards a proximal lesion at L2/3 alongside an otherwise “unexplainable” L5 deficit. EMG studies highlighted a pre-ganglionic lesion with normal sensory conduction, which highlighted a chronic right L5 radiculopathy. With the clinical findings and the imaging being discordant (as no compression at L4/5 was noted) and the immediate postoperative improvement of the foot drop, it further supported the theory of intrathecal compression of the L5 nerve as it descends within the cauda equina.

Although the patient was counselled that his chronic, painless foot drop was unlikely to improve, he experienced complete postoperative resolution. This outcome is remarkable given that recent meta-analyses have noted poor prognoses for patients with painless foot drop or those with an MRC grade < 2/5 [10,11].

Thus, surgical decompression should not be withheld based on the chronicity of the foot drop or lack of pain alone, as this case demonstrates that significant recovery remains possible.

## Conclusions

This case highlights one of the 'great masqueraders' in spine surgery: a false localising sign due to a lumbar spinal stenosis. While degenerative lumbar spinal stenosis typically follows a predictable dermatomal pattern, L2/3 stenosis can present with both typical and atypical features in the same setting, as in our case with an unexpected L5 foot drop. Although EMG studies are invaluable when clinical findings and imaging appear discordant, it is imperative that clinicians recognise proximal thecal sac compression as a viable aetiology for distal deficits. Understanding the topographical arrangement of the cauda equina allows for more nuanced patient counselling regarding the goals of surgery and the potential for neurological recovery, even in chronic cases.
